# PLA Electrospun Scaffolds for Three-Dimensional Triple-Negative Breast Cancer Cell Culture

**DOI:** 10.3390/polym11050916

**Published:** 2019-05-23

**Authors:** Emma Polonio-Alcalá, Marc Rabionet, Xavier Gallardo, David Angelats, Joaquim Ciurana, Santiago Ruiz-Martínez, Teresa Puig

**Affiliations:** 1 New Therapeutic Targets Laboratory (TargetsLab)—Oncology Unit, Department of Medical Sciences, Faculty of Medicine, University of Girona, Emili Grahit 77, 17003 Girona, Spain; emma.polonio@udg.edu (E.P.-A.); m.rabionet@udg.edu (M.R.); xagallardo@gmail.com (X.G.); dangelatslobo4@gmail.com (D.A.); 2 Product, Process and Production Engineering Research Group (GREP), Department of Mechanical Engineering and Industrial Construction, University of Girona, Maria Aurèlia Capmany 61, 17003 Girona, Spain; quim.ciurana@udg.edu

**Keywords:** breast cancer, triple-negative breast cancer, three-dimensional cell culture, poly(lactic acid), EGFR, STAT3

## Abstract

Three-dimensional (3D) systems provide a suitable environment for cells cultured in vitro since they reproduce the physiological conditions that traditional cell culture supports lack. Electrospinning is a cost-effective technology useful to manufacture scaffolds with nanofibers that resemble the extracellular matrix that surround cells in the organism. Poly(lactic acid) (PLA) is a synthetic polymer suitable for biomedical applications. The main objective of this study is to evaluate electrospun (ES)-PLA scaffolds to be used for culturing cancer cells. Triple-negative breast cancer (TNBC) is the most aggressive breast cancer subtype with no validated targeted therapy and a high relapse rate. MDA-MB-231 TNBC cells were grown in scaffolds from two different PLA concentrations (12% and 15% *w*/*v*). The appropriateness of ES-PLA scaffolds was evaluated using a cell proliferation assay. EGFR and STAT3 gene expression and protein levels were compared in cells grown in 2D versus in 3D cultures. An increase in STAT3 activation was shown, which is related to self-renewal of cancer stem cells (CSCs). Therefore, the enrichment of the breast CSC (BCSC) population was tested using a mammosphere-forming assay and gene expression of BCSC-related stemness and epithelial-to-mesenchymal transition markers. Based on the results obtained, ES-PLA scaffolds are useful for 3D cultures in short culture periods with no BCSC-enrichment.

## 1. Introduction

Two-dimensional (2D) cell culture is a well-established system used in the vast majority of studies involving cells. However, this approach is not completely faithful to reality since in the organism, cells are surrounded by the extracellular matrix (ECM), which provides a physical support for cells; this is missing in in vitro flat conditions. The ECM has also been proven to exert an important role in many cellular processes, such as cell growth or differentiation [1,2]. Monolayer cell cultures hardly mimic the physiological architecture and environment [3]. Therefore, three-dimensional (3D) cell culture has become an important hotspot in worldwide research in order to fix the aforementioned issues [4].

Researchers have proved that 3D systems provide a suitable spatial distribution for cells cultured in vitro, allowing cell–cell and cell–matrix interactions [5]. Hence, 3D-cultured cells have a different morphology, proliferation rate, gene expression and protein synthesis, differentiation, and drug metabolism compared to 2D culture [6,7,8,9,10]. Scaffold-based culture is a type of 3D culture that has been increasingly gaining importance in medical research, especially in oncology. Electrospinning and Fused Filament Fabrication (FFF) techniques are the two most used methods nowadays to manufacture these structures [11]. Using the electrospinning, a more ECM-resembling scaffold within the micrometer to nanometer fibers range may be formed [12,13]. Electrospinning has been used for a wide range of applications, including drug delivery [14] or postoperative adhesion prevention [15]. Moreover, synthetic materials come with some advantages, such as high versatility and reproducibility [16]. Among them, poly(lactic acid) (PLA), a biodegradable synthetic polymer that has been declared to be biocompatible by the United States (US) Food and Drug Administration (FDA), is used in scaffold manufacturing, mostly with bone tissue engineering purposes such as in dentistry and orthopedics [17]. Furthermore, no immune rejection or cytotoxic effects were found in patients with PLA coronary stents implanted for six months [18]. To date, however, no studies have been published using electrospun (ES)-PLA scaffolds for oncogenic purposes.

Breast cancer is the most prevalent cancer among women in US and Europe. It is also the second leading cause of cancer death in women [19,20]. Between 15–20% of breast cancer cases correspond to triple-negative breast cancer (TNBC), an aggressive subtype with no overexpression of the estrogen receptor, progesterone receptor, or epidermal growth factor receptor-2 (HER2) [21,22]. The lack of known druggability leaves cytotoxic chemotherapy as the only possible treatment option for these patients. Despite a good initial response to chemotherapy, TNBC patients present a high relapse rate leading to poor prognosis, thus more effective therapies are needed [23,24].

In the current study, we aim to test whether TNBC cells can be cultured in ES-PLA fibrous scaffolds and analyze the impact of this 3D culture on cancer cells’ behaviour. In addition, stem features were assessed in order to corroborate a possible enrichment of the breast cancer stem cell (BCSC) population. Based on the results obtained, we conclude that ES-PLA scaffolds are suitable for in vitro 3D culture of the TNBC MDA-MB-231 cell line with no enlargement of BCSCs observed within the culture days tested.

## 2. Materials and Methods

### 2.1. Polymer

The polymer chosen to manufacture the scaffolds was poly(lactic acid) (PLA, BCN3D Technologies©, Barcelona, Spain), commonly used in 3D printing for bone tissue replacement. The selected solvent was chloroform (Labkem, Labbox Labware, Barcelona, Spain). PLA characteristics extracted from manufacture’s indications are shown in Table 1.

### 2.2. Polymer Thermal Analysis

The thermal properties of PLA samples (7.01 mg) were measured by Differential Scanning Calorimetry (DSC; TA-Q2000, Lukens Drive, New Castle, PA, USA). The melting point (Tm), glass-transition temperature (*T*_g_), and cold crystallization was measured from 25 to 180 °C under nitrogen in aluminum capsules at heating rate of 10 °C/min.

The thermal stability of the samples (9.25 mg) was investigated with thermogravimetric analysis (TGA; Mettler-Toledo TGA/DSC 1, Schwerzenbach, Switzerland) under nitrogen from 30 to 700 °C at a heating rate of 10 °C/min.

### 2.3. Electrospun Scaffolds Manufacturing

Two different PLA concentrations of 12% and 15% *w*/*v* were dissolved with chloroform at room temperature and under stirring. Scaffolds were manufactured by an electrospinning machine (Spraybase, Dublin, Ireland) using a 24 G needle emitter with an inner diameter of 0.55 mm. A fixed voltage of 7 kV and a flow rate of 2 mL/h was established by the Syringe Pump Pro software (New Era Pump Systems, Farmingdale, NY, USA). The collector was placed at 15 cm from the emitter. The electrospinning process was performed injecting 5 mL of the desired solution. The resulting scaffolds were cut using a scalpel into squares of 2.5 cm for their use in 6-well plates or of 1.6 cm for 12-well plates.

### 2.4. Scaffold Physical Characterization

12-well scaffolds manufactured using 12% and 15% PLA solution were weighed by Sartorius ED224S analytical balance (Sartorius, Göttingen, Germany) and scaffold thickness was measured using Mahr Micromar 40EWV (Mahr, Göttingen, Germany). Scanning electron microscopy (SEM; Zeiss, Oberkochen, Germany) was used to characterize the microarchitecture of the ES-PLA scaffolds. To discern the fiber uniformity, different images from the top and bottom sides were taken. Fiber diameter, surface porosity, and pore area were calculated from both sides to obtain the average value. The images were processed with the ImageJ software (National Institutes of Health, Bethesda, MD, USA). At least three scaffolds were tested.

### 2.5. Cell Line and Culture Conditions

MDA-MB-231 human TNBC cell line was obtained from the American Type Culture Collection (ATCC; Rockville, MD, USA). Cells were grown in Dublecco’s Modified Eagle’s Medium (DMEM), supplemented with 10% fetal bovine serum (FBS) and 50 U/mL of penicillin/streptomycin (HyClone, Logan, UT, USA). MDA-MB-231 cells were maintained at 37 °C and 5% CO_2_ atmosphere. Cells were monitored routinely and found to be mycoplasma-free. 

### 2.6. Three-Dimensional Cell Culture

PLA scaffolds were sterilized by submersion in a solution of 70% ethanol overnight, washed two times with phosphate-buffered saline (PBS, Hyclone), and exposed to UV light for 30 min with no alteration of the properties as previously described [25].

Sterilized scaffolds were placed in non-adherent cell culture 6- or 12-well plates (Sarstedt, Nümbrecht, Germany) and soaked in DMEM for 30 min at 37 °C and 5% CO_2_ humidified atmosphere prior to cell seeding with the aim to promote cell attachment. Then, the corresponding cell density was prepared into a reduced volume of medium (50 µL for 12-well scaffolds and 100 µL for 6-well ones) and pipetted drop by drop over the center of the scaffolds. Therefore, approximately the whole scaffold surface was covered with cell suspension and the scaffold remain soaked but with no cell loss over the well plate. Finally, seeded scaffolds were incubated for three hours to allow cell attachment at 37 °C and 5% CO_2_ atmosphere, then DMEM was added. Bidimensional (2D) cell culture was performed as a control in adherent cell culture microplates (Sartstedt) with the same cell density used in 3D culture.

### 2.7. Cell Proliferation Assay

To investigate cell proliferation, MDA-MB-231 cells were seeded into adherent 12-well plates for three and six days at a density of 50,000 and 8000 cells/well, respectively. Next, scaffolds were washed two times with PBS, PLA structures were placed in new wells to ensure only scaffold-attached cells would be analyzed, and a 3-(4,5-dimethylthiazolyl-2)-2,5-diphenyltetrazolium bromide (MTT) assay was performed as described elsewhere [26].

### 2.8. Quantitative Real-Time PCR Analysis

Suspensions of 125,000 and 20,000 MDA-MB-231 cells were seeded on standard wells and 15% PLA scaffolds were placed in non-adherent 6-well plates for three and six days, respectively. Then, scaffolds were washed twice with PBS and PLA structures were placed in new wells. MDA-MB-231 cells were detached as mentioned above. Trypsinized cells from 2D or 3D cultures were suspended with 750 µL of Qiazol (Qiagen, Hilden, Germany). The total RNA from each sample was isolated using an RNeasy Mini Kit (Qiagen) and reverse-transcribed into complementary DNA (cDNA) using the High Capacity cDNA Archive Kit (Applied Biosystems, Foster City, CA, USA). Gene expression levels were assessed using LightCycler 480 Real-Time PCR System (Roche, Basel, Switzerland) with LightCycler 480 SYBR Green I Master (Roche) as previously described [27]. Primers were designed as shown in Table 2.

Gene expression levels were quantified by the double delta Cycle threshold (Ct) analysis method and normalized to the housekeeping gene GAPDH [28].

### 2.9. Protein Analysis

A suspension of 125,000 and 20,000 MDA-MB-231 cells were seeded on 15% PLA scaffolds placed in 6-well plates and in adherent 6-well plates for three and six days, respectively. Next, scaffolds were washed twice with PBS and PLA meshes were placed in new wells. Cells were detached as described above. Cell pellets were lysed in ice-cold lysis buffer (Cell Signaling Technology, Inc., Danvers, MA, USA) with 100 mg/mL of the protease inhibitor phenylmethylsulfonylfluoride (PMSF) by vortexing every 5 min for 30 min. The same protein quantity (20 µg) was heated in lithium dodecyl sulfate (LDS) sample buffer with sample reducing agent (Invitrogen, Carlsbad, CA, USA) for 10 min at 70 °C, electrophoresed on acrylamide gels (Bio-Rad Laboratories, Inc., Hercules, CA, USA), and transferred onto nitrocellulose membranes (Thermo Fisher Scientific, Waltham, MA, USA). Transferred membranes were blocked (blocking buffer of 5% bovine serum albumin (BSA) in tris-buffered salineTBS 0.05% Tween (TBS-T)) at room temperature for 1 h. Then, membranes were incubated overnight with the following primary antibodies: Rabbit polyclonal antibodies against pEGFR^Y1068^ (Cell Signaling Technology Inc.; #2234S; dilution 1:1000), STAT3 (Cell Signaling Technology Inc.; #4904S; dilution 1:1000), pSTAT3^Y705^ (Cell Signaling Technology Inc.; #9131S; dilution 1:1000), rabbit monoclonal antibody against EGFR (Cell Signaling Technology Inc.; #4267S; dilution 1:1000), and mouse polyclonal antibody GAPDH (Proteintech, Manchester, UK; #60004-1-IG; dilution 1:50,000). Antibodies were diluted in blocking buffer at 4 °C. Specific horseradish peroxidase (HRP)-conjugated secondary antibody was incubated for 1 h at room temperature. Immune complexes were detected using a chemiluminescent HRP substrate (ClarityTM Western ECL Substrate (Bio-Rad Laboratories, Inc.)) and a ChemiDoc™ Imaging System (Bio-Rad Laboratories, Inc.). GAPDH was used as a control of protein loading. Protein analyses were repeated at least three times and the representative results are shown.

### 2.10. Mammosphere-Forming Assay

50,000 and 8000 cells were seeded on 15% PLA scaffolds placed in 12-well adherent cell culture plates for three and six days, respectively. Afterward, scaffolds were washed two times with PBS and placed in new wells. Cells were detached with trypsin-EDTA (HyClone) at 37 °C and 5% CO_2_ atmosphere. Detached cells were centrifuged 5 min at 1000 rpm. Cell pellets were resuspended in DMEM/F12 medium (HyClone) supplemented with 0.2% B27 (Gibco, Waltham, MA, USA), epidermal growth factor (EGF) and fibroblast growth factor (FGF) (final concentration of 20 ng/mL; Milteny Biotec, Bergisch Gladbach, Germany), 1% l-glutamine, and 1% sodium pyruvate. A suspension of 2000 cells/well was seeded onto non-adherent 6-well cell culture microplates (Sarstedt) and incubated for 7 days at 37 °C and 5% CO_2_. Afterward, mammospheres bigger than 50 µm were counted. The mammosphere-forming Index (MFI) was calculated for each culture condition following the next equation, as described in previous works [26,29].

### 2.11. Statistical Analysis

All data results are expressed as mean ± standard error of the mean (SEM) and were analyzed by Student’s t test. The P value is shown in results when significance was reached (*: *p* < 0.05; **: *p* <0.01; ***: *p* < 0.001). The results obtained were confirmed by at least three independent experiments.

## 3. Results

### 3.1. Thermal Characterization of PLA

**Figure 1:**
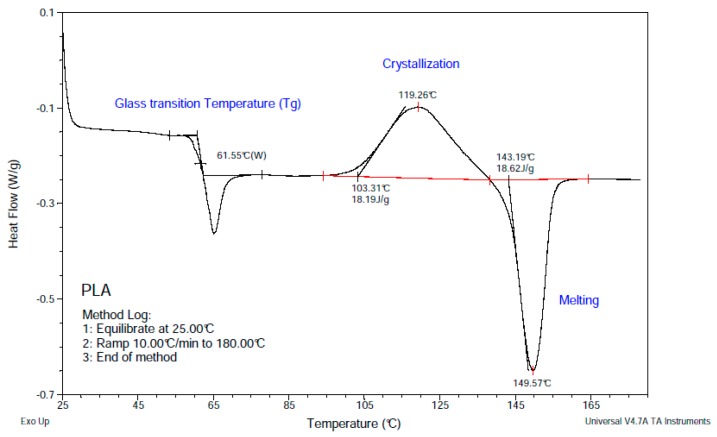
DSC curve of poly(lactic acid) (PLA).

**Figure 2:**
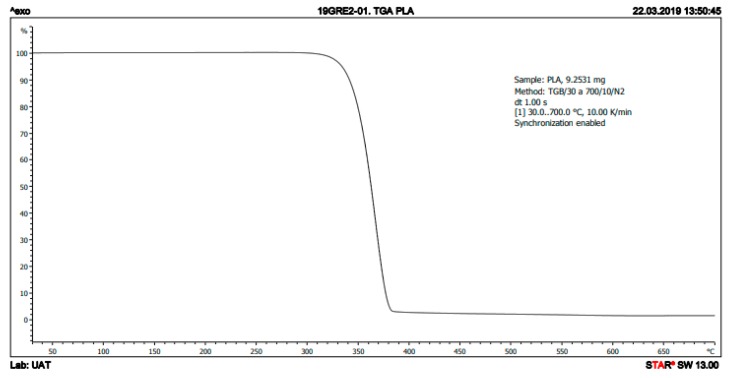
TGA curve of PLA.

The PLA filament’s thermal properties were studied through TGA and DSC. The DSC curve shows a *T*_g_ of 61.55 °C, crystallization at 119.26 °C, and a *T*_m_ of 149.57 °C (Figure 1). Furthermore, the TGA curve shows a complete degradation in one step between 310 and 380 °C (Figure 2).

### 3.2. Physical Characterization of ES-PLA Scaffolds

In an initial attempt to seek the proper scaffold to be used in 3D cell culture, different PLA-concentrations were used. Lower polymer concentrations than 12% led to electrospray instead of electrospinning. On the other hand, concentrations higher than 15% PLA resulted in electrospinning emitter obstruction (data not shown). Therefore, we decided to test the two possible extremes, i.e., 12% and 15% PLA. Once manufactured, the two different PLA-concentration scaffolds were cut into squares of 1.6 cm^2^ and then they were characterized by different approaches. Due to the similar quantity of polymer dissolved in the solutions to manufacture 12% and 15% ES-PLA scaffolds, no significant differences for weight or thickness values were found in any of the resulting scaffolds (Table 3).

**Figure 3:**
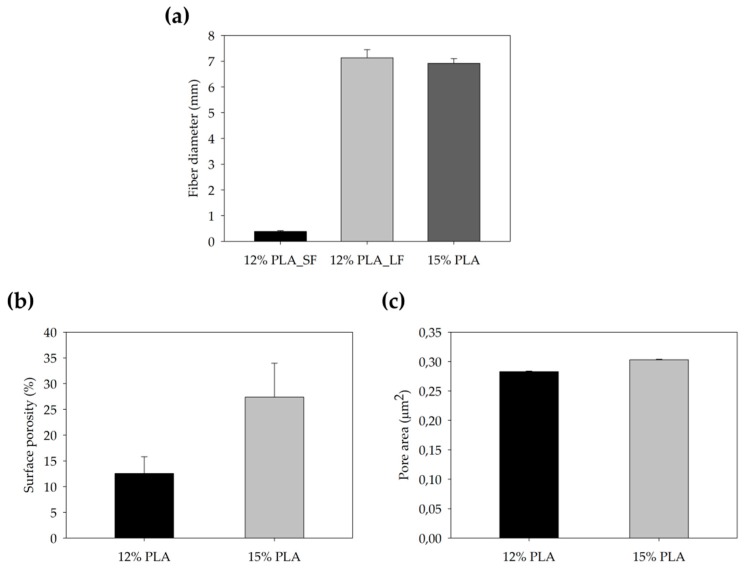
Features of 12% and 15% ES-PLA scaffolds. Images taken from the top and bottom sides were used to calculate (**a**) fiber diameter, (**b**) surface porosity, and (**c**) pore diameter. Results are shown as average ± SE. SF: small fibers; LF: large fibers.

Afterward, both structures were visualized by SEM to study the microscopic structure (Table 4). The uniformity of the fibers was confirmed through the visualization of the bottom and top sides. Scaffolds from 12% PLA showed two different types of filaments. One of them showed a diameter of roughly 7 µm, whereas the second type exhibited fibers nearly 20 times thinner. In contrast, 15% PLA scaffolds presented one filament population, lacking the slight ones (Figure 3). Both sides’ images from the lower PLA-concentration meshes revealed the possible presence of beads; this was not found in the higher PLA percentage on any side. Non-significant differences regarding the pore area or surface porosity were found between the two polymer concentrations, although it was observed that 15% PLA scaffolds had a non-significant higher surface porosity compared to 12% PLA scaffolds (Figure 3).

### 3.3. High Proliferation Rates are Obtained in Both 12% and 15% ES-PLA Scaffolds 

**Figure 4:**
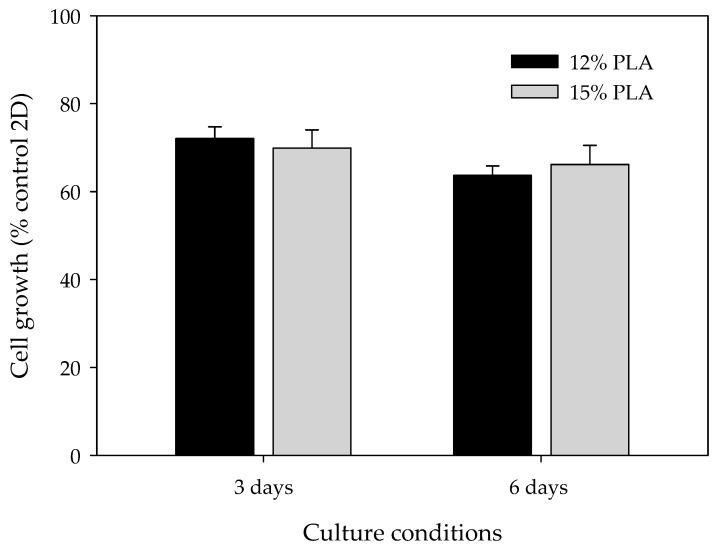
Cell proliferation analysis for MDA-MB-231 cells cultured in 3D using 12% PLA scaffolds (black bars) or 15% PLA scaffolds (grey bars) over three days (left) or six days (right). MDA-MB-231 cells cultured in 2D were used as a control. Experiments were performed at least three times.

Differences among microscopic features due to the percentage of PLA used may influence cell adhesion and growth. Therefore, cell viability assays were performed to ascertain whether MDA-MB-231 cells can be cultured using ES-PLA scaffolds and determine the optimal PLA concentration (Figure 4). Two culture periods were chosen, i.e., three and six days. As expected, cells cultured in 3D presented a lower proliferation rate than cells growing in a monolayer. Accordingly, these differences were more noticeable after six days. Nonetheless, cell growth was surprisingly high in both 12% and 15% PLA scaffolds, with no significant differences in any of the times assayed. Henceforth, the experiments were carried out using the non-beading 15% concentration PLA meshes.

### 3.4. EGFR and STAT3 are Altered in 3D-Cultured Cells

**Figure 5:**
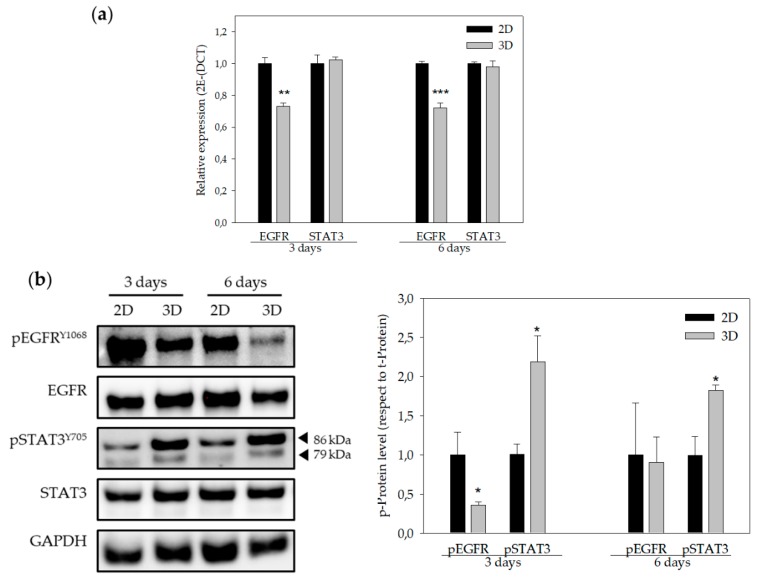
EGFR and STAT3 genes expression and protein levels. (**a**) EGFR and STAT3 gene expression of MDA-MB-231 cells cultured in 2D (black bars) and in 3D (grey bars) using 15% PLA scaffolds over three days (left) or six days (right). (**b**) Quantification of protein levels (EGFR and STAT3) obtained by Western blot analyses (right panel) of MDA-MB-231 cells cultured in 2D (black bars) and in 3D (grey bars) using 15% PLA scaffolds over three days (left) or six days (right). Results are expressed as the ratio of activated protein (p-Protein) vs. total levels of protein (t-Protein). Experiments were performed at least three times. * (*p* < 0.05) indicates levels of statistical significance. For an easier interpretation, the value obtained for cells cultured in 2D was adjusted to 1 and that of the 3D-cultured cells was calculated based on this.

Previous investigations have demonstrated that EGFR gene expression, as well as STAT3 and EGFR protein activation, was altered in 3D-cultured cells [30,31]. For this reason, EGFR and STAT3 gene and protein levels were assessed by quantitative real time PCR and Western blotting (Figure 5). As expected, EGFR gene expression levels were significantly lower in 3D-cultured cells compared to cells cultured in monolayer. However, no changes in STAT3 gene expression were visualized in the three or six day cultures when comparing 2D and 3D cultures (Figure 5a). Furthermore, EGFR protein phosphorylation levels were decreased, whereas phosphorylation levels of STAT3 increased in 3D culture conditions, both in three and six day cultures (Figure 5b).

Moreover, it has been demonstrated that the STAT3 pathway is involved in cancer stem cell (CSC) self-renewal and cancer chemoresistance [29,30]. Hence, ES-PLA scaffolds’ capacity to enrich the breast CSC (BCSC) population was evaluated.

### 3.5. ES-PLA Scaffolds Do Not Increase BCSC Population

Previously, our research group set up the conditions of the ES-scaffolds using polycaprolactone (PCL) to enrich the BCSC population in MDA-MB-231 cells [26,32]. BCSCs are a small subpopulation of cells capable of self-renewal, giving rise to the bulk of the tumour and responsible for relapse due to their chemoresistance [33,34,35]. We aimed then to discern the impact of ES-PLA scaffold culture on BCSC niche.

**Figure 6:**
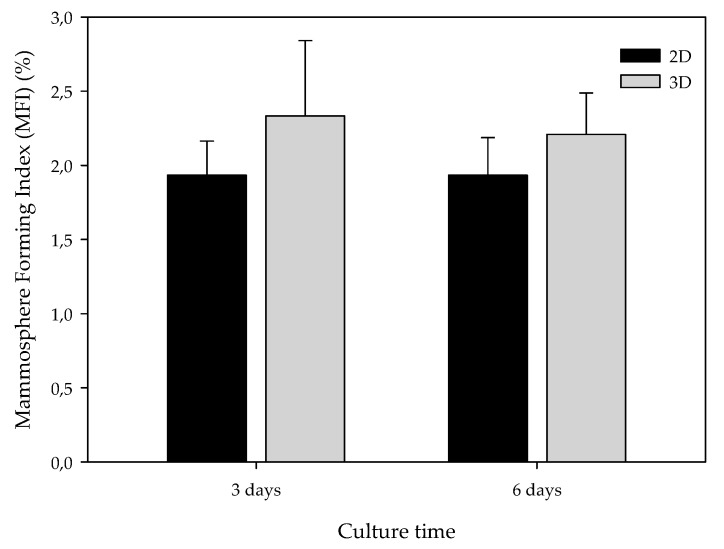
Mammosphere-forming Index (MFI) of MDA-MB-231 TNBC cells after monolayer (2D) or 15% PLA scaffolds (3D) culture. Experiments were performed at least three times.

The BCSC population also exhibits the capability to grow in non-adherent surfaces, proliferating into suspension spheres called mammospheres [36]. Taking advantage of this capability, the mammosphere-forming (MF) assay was performed in cells previously cultured in monolayer and 15% ES-PLA meshes. Different cell culture periods, three and six days, were also assessed. As exhibited in Figure 6 monolayer-cultured cells displayed MFI values close to 2% in both culture periods. In contrast, cells from 3D cultures showed a slightly higher MF capacity compared with its correspondent 2D control, although no significant differences were found. This trend was found in both culture days tested.

**Figure 7:**
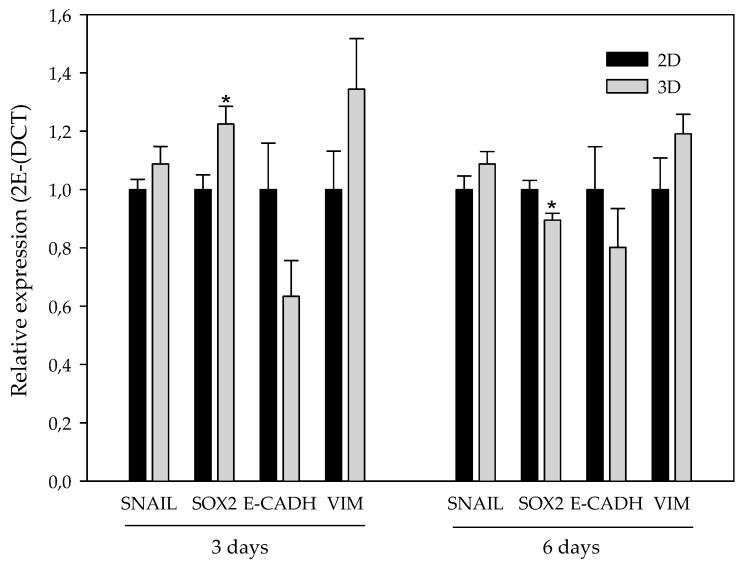
Epithelial-to-mesenchymal transition (EMT)-related and stemness gene expression. Stemness (Sox2) and EMT-related (Snail, E-cadherin, and Vimentin) gene expression of MDA-MB-231 culture in 2D (black bars) and in 3D (grey bars) using 15% PLA ES scaffolds over three days (left) and six days (right). Experiments were performed at least three times. For an easier interpretation, the value obtained for cells cultured in 2D was adjusted to 1 and that of the 3D-cultured cells was calculated based on this. * (*p* < 0.05) indicates levels of statistical significance.

CSCs showed higher expression of some stem cell markers, including Sox2 [37]. The role of epithelial-to-mesenchymal transition (EMT) has been related to the stem features acquisition [38,39,40]. EMT comprises different molecular alterations, including transcription factors such as Snail (zinc-finger protein SNAI1), among others, which modulate mesenchymal proteins like Vimentin (Vim) and downregulate epithelial proteins such as E-cadherin (E-cadh) [41]. EMT confers stem traits in non-stem cancer cells, possibly leading to BCSC enrichment during 3D culturing. For this reason, we evaluated the RNA expression of Snail, E-cadh, Vim, and Sox2 on 2D- and 3D-cultured cells (Figure 7).

Regarding EMT-related genes, the transcription factor Snail and the mesenchymal protein Vim presented a subtle trend to be upregulated in 3D-cultured cells with no significance. In addition, the observed tendency of Vim was higher after three days. In contrast, the expression of the epithelial protein E-cadh presented a non-significant decrease when comparing 3D-cultured cells with the monolayer culture. Again, the aforementioned decrease also exhibited a stronger trend after three days of culturing. Sox2 was significantly upregulated on cells cultured in scaffolds over three days, whereas it presented a downregulation at six days of culture.

## 4. Discussion

Despite the numerous advantages of traditional cell cultures in the field of cancer research, the monolayer growth of cells lacks a large number of tumor characteristics, including interactions with the ECM. This interactivity with non-cellular elements not only affects the morphological organization but also the activation of signaling pathways or the expression of proteins which may be biologically relevant [42]. In order to better understand the tumor behavior and improve the development of new therapeutic drugs, in vitro cells cultured in 3D systems will undoubtedly be of help. In this sense, ES-polymeric scaffolds have emerged as a cost-effective alternative to grow in vitro cells in 3D [43,44]. In this work, we studied for the first time the polymer PLA to manufacture scaffolds through ES for 3D culturing of breast cancer cells. The chosen PLA was first characterized in order to confirm its purity. Obtained DSC values, including glass transition, crystallization and melting temperature, were in concordance with those presented by Zou et al. [45]. Regarding the values showed in Table 1, the experimental PLA *T*_g_ was in agreement with the data provided by the manufacturer, whereas the obtained Tm was slightly lower. Moreover, the TGA curve acquired showed the same distribution in one step and temperatures than the TGA described in Zou et al. [45]. Consequently, the PLA used to electrospin scaffolds showed an optimal purity.

Both scaffolds from 12% and 15% PLA solution displayed similar weight and thickness values, due to the little difference of material amount of each solution. Moreover, they exhibited comparable filament diameter and porosity. Interestingly, 12% PLA meshes also possessed a secondary filament population, with a lower average diameter of 385 nm, as well as the presence of a non-filamented material known as beads. Previous works also observed the aforementioned phenomenon. For instance, Damanik et al. electrospun a 15% PLA solution at 12 kV and 7.5 mL/h, resulting in PLA meshes with a filament diameter close to 4.5 µm as well as the presence of a minor, thinner filament population [46]. The presence of beads was widely related to the use of a low polymer concentration [26,47,48].

3D-cultured cells are known to proliferate more slowly and survive for a longer period of time than 2D-cultured cells [49]. These microarchitecture differences in the PLA scaffolds did not directly affect MDA-MB-231 proliferation rate, with values around 70% compared to monolayer culture. The same cell line was previously cultured on PLA scaffolds made by FFF technology, resulting in larger pores and filament diameters. In this case, MDA-MB-231 cell proliferation was much lower, ranging from 5% to 25% compared to 2D-cultured cells, with significant differences between scaffold designs [32]. The improvement of cell proliferation into ES-PLA scaffolds makes them an optimal device for in vitro 3D cell culture, reproducing the spatial architecture of tumors.

EGFR signaling transduction pathways are related to cell proliferation, metabolism, migration, and survival [50]. Approximately 45% of TNBC patients show a higher expression of EGFR [51]. The results obtained showed a significant decrease in EGFR gene expression. Moreover, phosphorylation levels were significantly reduced after three days, whereas no change in total protein levels was noticed in the 3D cultures. Howes et al. observed that EGFR protein phosphorylation decreased whereas EGFR total protein levels increased in BT-474 Luminal B breast carcinoma cells co-cultured with human fibroblast and endothelial cells in 3D cultures compared to monolayer cultures [30]. Ekert et al. demonstrated that different lung adenocarcinoma cell lines showed a reduction of EGFR total protein levels in 3D cultures compared to monolayer cultures. In addition, EGFR phosphorylation levels decreased when cells were stimulated with growth factors in 3D cultures [52]. Unlike cells cultured using 3D systems, monolayer-cultured cells are directly exposed to a medium rich in growth factors that might stimulate EGFR expression.

STAT3 is a transcription factor which controls the expression of different genes involved in tumor progression [53]. Despite STAT3 gene expression being similar in both cultures’ systems, MDA-MB-231 cells exhibited an increase in STAT3 phosphorylation levels when cultured in ES-PLA scaffolds. This result is in agreement with that of Leslie et al., who demonstrated that mammary epithelial cells transformed with H-rasV12 and, when grown in mouse tissues, resulted in higher STAT3 phosphorylation levels compared to monolayer cultures [31], suggesting that STAT3 has a role in a 3D environment. Furthermore, it has been demonstrated that the STAT3 pathway is involved in CSCs self-renewal. Kroon et al. observed that STAT3 signaling was related to clonogenic and tumorigenic potential of CSCs in prostate cancer [54]. Also, Lin et al. showed that STAT3 was related to tumor growth and tumor-initiating potential in colon cancer [55].

According to these results, we wanted to discern whether ES-PLA scaffolds at the conditions assayed favored the maintenance of the BCSC population. CSCs have the capability to grow in suspension, forming mammospheres and showing greater expression of some stem cell markers, including Sox2 and OCT3/4 [36,37]. CSCs are related to the EMT process, a well-regulated cell program in embryogenesis which is responsible for much of tissue development [36]. Cancer epithelial cells can lose epithelial cell polarity and acquire mesenchymal abilities, such as invasion and migration, through EMT [56]. Hence, EMT confers stem traits, possibly leading to BCSC-enrichment in 3D cultures. Consequently, the capacity to form mammospheres, as well as the expression of genes related to EMT and stemness, was assessed. Cells previously cultured in scaffolds exhibited a subtle, non-significant, higher MF Index value, with a maximum at day three of culturing. Interestingly, it was also at this incubation period when significant overexpression of the Sox2 stemness marker was found on 3D-cultured cells. Likewise, expression of mesenchymal markers Snail and Vim on cells cultured in scaffolds exhibited uniformity or a non-significant upregulation, respectively, when compared with the monolayer control. Therefore, cells cultured into ES-PLA scaffolds seem to maintain a more epithelial phenotype rather than acquiring mesenchymal and more invasive features of CSCs. It is noteworthy that expression of the epithelial protein E-cadherin, which can be repressed by some transcription factors, including Snail, to induce EMT [41,56], seems to be downregulated on cells cultured in scaffolds at three days.

## 5. Conclusions

The high cell proliferation rates obtained using ES-PLA scaffolds make them a useful tool for 3D TNBC cell culture, taking the in vitro culture a step closer to physiological conditions and facilitating the study within a 3D environment of this aggressive cancer subtype devoid of a validated targeted therapy. In this sense, scaffolds may fill in the large gap between in vitro cell culture and in vivo experimentation, which could be useful for applications such as drug screening. Despite the fact that some stem features tend to be empowered on 3D-cultured cells, ES-PLA scaffolds do not show an important CSC-enrichment at the conditions assayed. However, taken altogether, these results set up the basis for future studies using ES-PLA scaffolds for oncology research purposes.

## Figures and Tables

**Table 1:** Poly(lactic acid) properties.

Molecular Weight (g/mol)	Melting Point (°C)	Glass Transition (°C)	Young’s Modulus (MPa)	Strain at Break (%)	Degradation Time (Months)
30,000	173–178	60–65	108	3.5%	12

**Table 2:** Forward and reverse sequences of analyzed genes.

Gene	Forward sequence (5′-3′)	Reverse sequence (5′-3′)
*SNAIL*	GCTGCAGGACTCTAATCCAGA	ATCTCCGGAGGTGGGATG
*SOx2*	AACCCCAAGATGCACAACTC	GCTTAGCCTCGTCGATGAAC
*E-cadh*	TGGAGGAATTCTTGCTTTGC	CGCTCTCCTCCGAAGAAAC
*VIM*	TGGTCTAACGGTTTCCCCTA	GACCTCGGAGCGAGAGTG
*EGFR*	CATGTCGATGGACTTCCAGA	GGGACAGCTTGGATCACACT
*STAT3*	CACCTTCAGGATGTCCGGAA	ATCCTGGAGATTCTCTACCACTTTCA
*GAPDH*	TCTTCCAGGAGCGAGATC	CAGAGATGATGACCCTTTTG

**Table 3:** Weight and thickness of 12% and 15% PLA scaffolds.

Scaffold trait	12%	15%
Weight (mg)	5.83 ± 0.63	6.23 ± 0.44
Thickness (µm)	68.33 ± 6.89	68.00 ± 3.51

**Table 4:** Microscopic characterization of 12% and 15% electrospun (ES)-PLA scaffolds (7 kV, 5 mL/h). Micrographs from the top and bottom sides at different magnifications were visualized using SEM.

**12% PLA**	Side	Magnification		
		200×	1500×	5000×
	Top	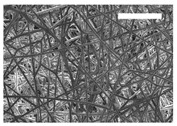	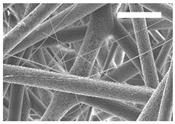	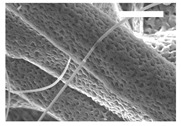
	Bottom	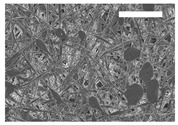	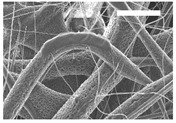	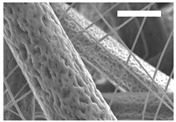
**15% PLA**	Top	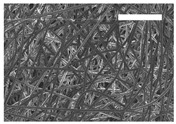	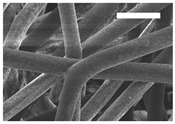	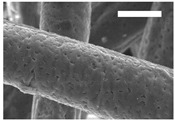
	Bottom	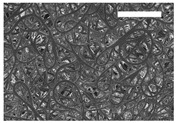	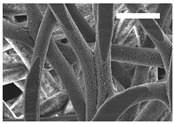	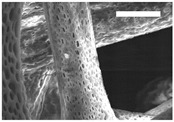
		Scale bars: 150 µm	Scale bars: 20 µm	Scale bars: 6 µm

## References

[1] Frantz C., Stewart K.M., Weaver V.M. (2010). The extracellular matrix at a glance. J. Cell Sci..

[2] Theocharis A.D., Skandalis S.S., Gialeli C., Karamanos N.K. (2016). Extracellular matrix structure. Adv. Drug Deliv. Rev..

[3] Abbott A. (2003). Cell culture: Biology’s new dimension. Nature.

[4] Weigelt B., Ghajar C.M., Bissell M.J. (2014). The need for complex 3D culture models to unravel novel pathways and identify accurate biomarkers in breast cancer. Adv. Drug Deliv. Rev..

[5] DesRochers T.M., Palma E., Kaplan D.L. (2014). Tissue-engineered kidney disease models. Adv. Drug Deliv. Rev..

[6] Pampaloni F., Stelzer E.H.K., Leicht S., Marcello M. (2010). Madin-Darby canine kidney cells are increased in aerobic glycolysis when cultured on flat and stiff collagen-coated surfaces rather than in physiological 3-D cultures. Proteomics.

[7] Loessner D., Stok K.S., Lutolf M.P., Hutmacher D.W., Clements J.A., Rizzi S.C. (2010). Bioengineered 3D platform to explore cell-ECM interactions and drug resistance of epithelial ovarian cancer cells. Biomaterials.

[8] Ridky T.W., Chow J.M., Wong D.J., Khavari P.A. (2010). Invasive three-dimensional organotypic neoplasia from multiple normal human epithelia. Nat. Med..

[9] Li C.-L., Tian T., Nan K.-J., Zhao N., Guo Y.-H., Cui J., Wang J., Zhang W.-G. (2008). Survival advantages of multicellular spheroids vs. monolayers of HepG2 cells in vitro. Oncol. Rep..

[10] Chopra V., Dinh T.V., Hannigan E.V. (1997). Three-dimensional endothelial-tumor epithelial cell interactions in human cervical cancers. In Vitro Cell. Dev. Biol. Anim..

[11] Polonio-Alcalá E., Rabionet M., Ruiz-Martínez S., Ciurana J., Puig T. (2018). Three-Dimensional Manufactured Supports for Breast Cancer Stem Cell Population Characterization. Curr. Drug Targets.

[12] Sill T.J., von Recum H.A. (2008). Electrospinning: Applications in drug delivery and tissue engineering. Biomaterials.

[13] Knight E., Przyborski S. (2014). Advances in 3D cell culture technologies enabling tissue-like structures to be created in vitro. J. Anat..

[14] Sun Y., Shan H., Wang J., Wang X., Yang X., Ding J. (2019). Laden Nanofiber Capsules for Local Malignancy Chemotherapy. J. Biomed. Nanotechnol..

[15] Zhang J., Liu H., Xu H., Ding J.-X., Zhuang X.-L., Chen X.-S., Chang F., Xu J.-Z., Li Z.-M. (2014). Molecular weight-modulated electrospun poly(ε-caprolactone) membranes for postoperative adhesion prevention. RSC Adv..

[16] Liu X., Holzwarth J.M., Ma P.X. (2012). Functionalized Synthetic Biodegradable Polymer Scaffolds for Tissue Engineering. Macromol. Biosci..

[17] Gregor A., Filová E., Novák M., Kronek J., Chlup H., Buzgo M., Blahnová V., Lukášová V., Bartoš M., Nečas A., Hošek J. (2017). Designing of PLA scaffolds for bone tissue replacement fabricated by ordinary commercial 3D printer. J. Biol. Eng..

[18] Tamai H., Igaki K., Kyo E., Kosuga K., Kawashima A., Matsui S., Komori H., Tsuji T., Motohara S., Uehata H. (2000). Initial and 6-month results of biodegradable poly-l-lactic acid coronary stents in humans. Circulation.

[19] Ferlay J., Steliarova-Foucher E., Lortet-Tieulent J., Rosso S., Coebergh J.W.W., Comber H., Forman D., Bray F. (2013). Cancer incidence and mortality patterns in Europe: Estimates for 40 countries in 2012. Eur. J. Cancer.

[20] Siegel R.L., Miller K.D., Jemal A. (2018). Cancer statistics, 2018. CA. Cancer J. Clin..

[21] Dent R., Trudeau M., Pritchard K.I., Hanna W.M., Kahn H.K., Sawka C.A., Lickley L.A., Rawlinson E., Sun P., Narod S.A. (2007). Triple-negative breast cancer: Clinical features and patterns of recurrence. Clin. Cancer Res..

[22] Anders C.K., Carey L.A. (2009). Biology, Metastatic Patterns, and Treatment of Patients with Triple-Negative Breast Cancer. Clin. Breast Cancer.

[23] Liedtke C., Rody A., Gluz O., Baumann K., Beyer D., Kohls E.-B., Lausen K., Hanker L., Holtrich U., Becker S. (2015). The prognostic impact of age in different molecular subtypes of breast cancer. Breast Cancer Res. Treat..

[24] Cinkaya A., Akin M., Sengul A. (2016). Evaluation of treatment outcomes of triple-negative breast cancer. J. Cancer Res. Ther..

[25] Guerra A.J., Cano P., Rabionet M., Puig T., Ciurana J. (2018). Effects of different sterilization processes on the properties of a novel 3D-printed polycaprolactone stent. Polym. Adv. Technol..

[26] Rabionet M., Yeste M., Puig T., Ciurana J. (2017). Electrospinning PCL scaffolds manufacture for three-dimensional breast cancer cell culture. Polymers (Basel)..

[27] Giró-Perafita A., Palomeras S., Lum D.H., Blancafort A., Viñas G., Oliveras G., Pérez-Bueno F., Sarrats A., Welm A.L., Puig T. (2016). Preclinical Evaluation of Fatty Acid Synthase and EGFR Inhibition in Triple Negative Breast Cancer. Clin. Cancer Res..

[28] Livak K.J., Schmittgen T.D. (2001). Analysis of Relative Gene Expression Data Using Real- Time Quantitative PCR and the 2(-Delta Delta C(T)) Method. METHODS.

[29] Giró-Perafita A., Rabionet M., Puig T., Ciurana J. (2016). Optimization of Poli(Ɛ-caprolactone) scaffolds suitable for 3D cancer cell culture. Procedia CIRP.

[30] Howes A.L., Richardson R.D., Finlay D., Vuori K. (2014). 3-Dimensional culture systems for anti-cancer compound profiling and high-throughput screening reveal increases in EGFR inhibitor-mediated cytotoxicity compared to monolayer culture systems. PLoS ONE.

[31] Leslie K., Gao S.P., Berishaj M., Podsypanina K., Ho H., Ivashkiv L., Bromberg J. (2010). Differential interleukin-6/Stat3 signaling as a function of cellular context mediates Ras-induced transformation. Breast Cancer Res..

[32] Polonio-Alcalá E., Rabionet M., Guerra A., Yeste M., Ciurana J., Puig T., Polonio-Alcalá E., Rabionet M., Guerra A.J., Yeste M. (2018). Screening of Additive Manufactured Scaffolds Designs for Triple Negative Breast Cancer 3D Cell Culture and Stem-Like Expansion. Int. J. Mol. Sci..

[33] Palomeras S., Ruiz-Martínez S., Puig T. (2018). Targeting Breast Cancer Stem Cells to Overcome Treatment Resistance. Molecules.

[34] Diehn M., Cho R.W., Lobo N.A., Kalisky T., Dorie M.J., Kulp A.N., Qian D., Lam J.S., Ailles L.E., Wong M. (2009). Association of reactive oxygen species levels and radioresistance in cancer stem cells. Nature.

[35] Nakamura K., Iinuma H., Aoyagi Y., Shibuya H., Watanabe T. (2010). Predictive value of cancer stem-like cells and cancer-associated genetic markers for peritoneal recurrence of colorectal cancer in patients after curative surgery. Oncology.

[36] Dontu G., Abdallah W.M., Foley J.M., Jackson K.W., Clarke M.F., Kawamura M.J., Wicha M.S. (2003). In vitro propagation and transcriptional profiling of human mammary stem / progenitor cells. Genes Dev..

[37] Okumura-Nakanishi S., Saito M., Niwa H., Ishikawa F. (2005). Oct-3/4 and Sox2 Regulate *Oct-3/4* Gene in Embryonic Stem Cells. J. Biol. Chem..

[38] Kotiyal S., Bhattacharya S. (2014). Breast cancer stem cells, EMT and therapeutic targets. Biochem. Biophys. Res. Commun..

[39] Luo M., Brooks M., Wicha M.S. (2015). Epithelial-mesenchymal plasticity of breast cancer stem cells: Implications for metastasis and therapeutic resistance. Curr. Pharm. Des..

[40] Mani S.A., Guo W., Liao M.-J., Eaton E.N., Ayyanan A., Zhou A.Y., Brooks M., Reinhard F., Zhang C.C., Shipitsin M. (2008). The Epithelial-Mesenchymal Transition Generates Cells with Properties of Stem Cells. Cell.

[41] Moreno-Bueno G., Portillo F., Cano A. (2008). Transcriptional regulation of cell polarity in EMT and cancer. Oncogene.

[42] Xu X., Farach-Carson M.C., Jia X. (2014). Three-dimensional in vitro tumor models for cancer research and drug evaluation. Biotechnol. Adv..

[43] Palomeras S., Rabionet M., Ferrer I., Sarrats A., Garcia-Romeu M.L., Puig T., Ciurana J. (2016). Breast Cancer Stem Cell Culture and Enrichment Using Poly(ϵ-Caprolactone) Scaffolds. Molecules.

[44] Feng S., Duan X., Lo P.-K., Liu S., Liu X., Chen H., Wang Q. (2013). Expansion of breast cancer stem cells with fibrous scaffolds. Integr. Biol. (Camb)..

[45] Zou W., Chen R., Zhang H., Qu J. (2016). Preparation, melting behavior and thermal stability of poly(lactic acid)/poly(propylene carbonate) blends processed by vane extruder. AIP Conference Proceedings.

[46] Damanik F.F.R., Spadolini G., Rotmans J., Farè S., Moroni L. (2019). Biological activity of human mesenchymal stromal cells on polymeric electrospun scaffolds. Biomater. Sci..

[47] Fong H., Chun I., Reneker D. (1999). Beaded nanofibers formed during electrospinning. Polymer (Guildf)..

[48] Chen M., Patra P.K., Warner S.B., Bhowmick S. (2006). Optimization of electrospinning process parameters for tissue engineering scaffolds. Biophys. Rev. Lett..

[49] Li S., Lao J., Chen B.P., Li Y.S., Zhao Y., Chu J., Chen K.D., Tsou T.C., Peck K., Chien S. (2003). Genomic analysis of smooth muscle cells in 3-dimensional collagen matrix. FASEB J..

[50] Citri A., Yarden Y. (2006). EGF–ERBB signalling: Towards the systems level. Nat. Rev. Mol. Cell Biol..

[51] Nielsen T.O., Hsu F.D., Jensen K., Cheang M., Karaca G., Hu Z., Hernandez-Boussard T., Livasy C., Cowan D., Dressler L. (2004). Immunohistochemical and Clinical Characterization of the Basal-Like Subtype of Invasive Breast Carcinoma. Clin. Cancer Res..

[52] Ekert J.E., Johnson K., Strake B., Pardinas J., Jarantow S., Perkinson R., Colter D.C. (2014). Three-dimensional lung tumor microenvironment modulates therapeutic compound responsiveness in vitro--implication for drug development. PLoS ONE.

[53] Yu H., Lee H., Herrmann A., Buettner R., Jove R. (2014). Revisiting STAT3 signalling in cancer: New and unexpected biological functions. Nat. Rev. Cancer.

[54] Kroon P., Berry P.A., Stower M.J., Rodrigues G., Mann V.M., Simms M., Bhasin D., Chettiar S., Li C., Li P.-K. (2013). JAK-STAT Blockade Inhibits Tumor Initiation and Clonogenic Recovery of Prostate Cancer Stem-like Cells. Cancer Res..

[55] Lin L., Liu A., Peng Z., Lin H.J., Li P.K., Li C., Lin J. (2011). STAT3 Is Necessary for Proliferation and Survival in Colon Cancer-Initiating Cells. Cancer Res..

[56] Tomaskovic-Crook E., Thompson E.W., Thiery J.P. (2009). Epithelial to mesenchymal transition and breast cancer. Breast Cancer Res..

